# Mitochondrial Replacement: Ethics and Identity

**DOI:** 10.1111/bioe.12187

**Published:** 2015-10-19

**Authors:** Anthony Wrigley, Stephen Wilkinson, John B. Appleby

**Keywords:** mitochondrial replacement, genetics, identity, ethics, reproductive technology

## Abstract

Mitochondrial replacement techniques (MRTs) have the potential to allow prospective parents who are at risk of passing on debilitating or even life‐threatening mitochondrial disorders to have healthy children to whom they are genetically related. Ethical concerns have however been raised about these techniques. This article focuses on one aspect of the ethical debate, the question of whether there is any moral difference between the two types of MRT proposed: Pronuclear Transfer (PNT) and Maternal Spindle Transfer (MST). It examines how questions of identity impact on the ethical evaluation of each technique and argues that there is an important difference between the two. PNT, it is argued, is a form of therapy based on embryo modification while MST is, instead, an instance of selective reproduction. The article's main ethical conclusion is that, in some circumstances, there is a stronger obligation to use PNT than MST.

## 1. Introduction

Mitochondrial replacement techniques (MRTs) have the potential to allow prospective parents who are at risk of passing on mitochondrial disorders to have healthy children to whom they are genetically related. Mitochondrial disorders are often debilitating or even life‐shortening and the development of techniques to prevent them is a welcome prospect. Ethical concerns have however been raised about these techniques.^1^ This article focuses on just one aspect of the ethical debate, the question of whether there is any moral difference between the two types of MRT proposed: Pronuclear Transfer (PNT) and Maternal Spindle Transfer (MST). It examines how questions of identity impact on the ethical evaluation of each technique and argues that there is an important difference between the two. PNT, it is argued, is a form of therapy based on embryo modification while MST is, instead, an instance of selective reproduction. The paper's main ethical conclusion is that, in some circumstances, there may be a stronger obligation to utilise PNT than to use MST.

## 2. Mitochondrial Disorders and Mitochondrial Replacement Techniques

Mitochondria produce the energy needed for cellular functions. They also carry a genome (mtDNA) that is separate from the nuclear genome and is maternally inherited. If mutations occur in mtDNA, this can affect the ability of mitochondria to produce the energy needed for cellular functions. High concentrations of mutant mtDNA can result in a variety of mitochondrial disorders, which can cause severe suffering and premature death. One example of a devastating mitochondrial disorder is MELAS (Mitochondrial Encephalomyopathy, Lactic Acidosis and Stroke‐like episodes). The stroke‐like episodes caused by MELAS may result in loss of brain function, dementia, headaches, loss of vision, loss of muscular function, and seizures; the disease typically begins to present itself in childhood.^2^

Approximately one in every 6,500 children born in the UK has a mitochondrial disorder.^3^ The existing methods for avoiding mitochondrial disorders are predictive tests such as pre‐implantation genetic diagnosis (PGD) and pre‐natal diagnosis (PND). One difficulty with these, however, is that when parents receive adverse test results, their only option – if they want to avoid a mitochondrial disorder – is egg donation (or adoption). And while there are of course plenty of flourishing donor‐conceived families in existence, some people attach great value to having a child who is entirely genetically ‘theirs’. For these people, egg donation will be a seen as poor ‘second best’. In addition, in some countries, donor eggs are scarce, so that this option is further constrained.^4^ In an attempt to address this, two different mitochondrial replacement techniques (MRTs) are being developed.^5^

### (i) Maternal Spindle Transfer (MST)

The maternal spindle is a cluster of chromosomes that make up the egg's nuclear DNA. MST involves removing the maternal spindle from the mother's egg and placing it in an enucleated donor egg. This reconstructed egg contains the mother's nuclear DNA and a donor's healthy mitochondria. The egg is then fertilized so that it can develop into an embryo.

### (ii) Pronuclear Transfer (PNT)

PNT begins with two eggs: one from the mother, which contains diseased mitochondria, and a donor egg with healthy mitochondria. Both are fertilized and the two pronuclei (i.e. the respective genetic contributions from both the egg and sperm) are removed from each zygote. The enucleated zygote produced by the mother's egg and the father's sperm is then discarded. The two pronuclei that were created using the donor's egg and the father's sperm are also discarded. Next, the two pronuclei taken from the parents' embryo are injected into the enucleated ‘donor’ embryo. At the end of the procedure, the embryo produced contains the parents' nuclear DNA and the donor's healthy mitochondria.^6^

The UK Parliament recently approved *The Human Fertilisation and Embryology (Mitochondrial Donation) Regulations 2015* (HFER 2015) which further amend the Human Fertilisation and Embryology Act 1990 and, as of 29 October 2015, will make the UK the first country in the world to permit the licensed clinical use of MST and PNT on humans.^7^ However, the UK's Human Fertilization and Embryology Authority (HFEA) has stated that before either technique can be made available for clinical use, the HFEA will ‘develop a licensing framework through which applications can be considered from clinicians wanting to offer the two techniques set out in the regulations’ and ‘each application will be decided on a case by case basis and in accordance with the latest scientific advice.’^8^

## 3. Genes, Origins, and Identity

The connection between genes, biological origins, and identity is important for the ethical analysis of MRTs. There are several relevant ways in which these factors might determine identity, depending upon what sense of ‘identity’ is being employed.^9^

At the most fundamental level, genetic information has been seen as underpinning the numerical identity of persons.^10^ Person A and person B are *numerically* identical if and only if A and B are the very same human organism: if and only if A *is* B. Numerical identity concerns a very basic metaphysical means of determining questions of existence in terms of how we can refer to one and the same entity in any possible set of circumstances. In the case of persons, it allows us to determine *who* it is we are referring to at the most basic of levels. So, for example, we can say that President Barak Obama is *numerically identical* with the man who won the US presidential election in 2008 and what this means is that they (Obama and the 2008 presidential winner) are the very same entity – one and the same human being, not merely two people with a lot of similarities. This last aspect is important to emphasize in the case of numerical identity, as it allows us to keep reference to a particular individual fixed through a range of possible scenarios.

The challenge for an account of numerical identity is to explain what it is that determines the entity that we in fact are, despite the vast range of possible different properties and experiences we may or may not have. The explanation that our numerical identity is established in terms of our being a particular human organism is not fully satisfactory as it stands, as there are numerous ways our state as a human organism can be affected or changed that wouldn't themselves constitute a change in numerical identity. For example, various physical characteristics, such as height, weight, or even intelligence can be a result of environmental factors; we may lose parts of our body, for example the loss of limbs through accident, or even gain new parts, such as organs through transplantation. Although all of these might change or alter properties of our human organism, they are themselves all contingent – they might not have happened. As such, there needs to be a means of determining what it is that fixes our being the entity that we in fact are, in a way that is not open to contingent or accidental change. One of the most compelling accounts has been to provide an account of what it is to be the human organism that we are in terms of our biological origins, that is, our originating gametes.^11^ Hence it is a necessary feature of human organisms that we have the very same biological origins that we do, in fact, have, thereby giving us a causal account of our identity in terms of our originating genetic constitution. Therefore, I am the human organism I am in terms of my having the originating gametes I do, in fact, have. They are a necessary property of being me and anything that does not have the same originating gametes cannot be me.^12^

There are also other ways that genes determine a person's identity but we must be careful to distinguish the different senses of ‘identity’ in play. One major role is determining a person's ‘qualitative identity’ because they control, wholly or in part, many of our qualitative properties. These properties can be entirely physical, such as the nature of our chromosomes, or can interact with environmental factors to affect such things as our height, weight, intelligence, etc. Genes can also have implications for our ‘social identity’ insofar as social significance is placed on familial ancestry, genetic relatedness to other people, and future‐orientated concerns about continuing the family line.

Our focus in this article, though, is on numerical identity. This sense of identity plays an important role in debates about genetic selection techniques for future persons through Parfit's Non‐Identity Problem.^13^ In what follows, we argue that MRTs provide us with a new and interesting variation on the traditional Non‐Identity Problem.^14^

## 4. The Non‐Identity Problem and the MST/PNT Distinction

In the context of human reproduction, the Non‐Identity Problem arises most often because of the plausibility of what Parfit terms the Origin View. This says that:

… each person has this distinctive necessary property: that of having grown from the particular pair of cells from which this person in fact grew.^15^

If the Origin View (also termed ‘gametic essentialism’) is true then my existence is dependent, in a very strong sense, on the fact that a particular egg (E1) and particular sperm (S1) came together. Had they not – for example, had S2 fertilized E1 instead of S1 – then I would not have existed; someone else (a numerically distinct person) would have existed instead. We do not have space here to argue for the Origin View but merely note that it is widely regarded as a plausible principle and that its supposed truth underpins a great deal of the literature on the Non‐Identity Problem.^16^ Hence, we will be assuming its truth for the time being. The arguments that follow are, therefore, for the most part *conditional*, in that we are exploring what to think of the ethical differences between MST and PNT *on the assumption that* something like The Origin View is true. If the Origin View turned out to be false then things may have to be rethought.^17^

Gametic essentialism generates the Non‐Identity Problem. This problem is famously introduced by Parfit by way of an example.^18^ We are asked to imagine a woman who wishes to have a child. She knows that if she conceives now, her child will have a debilitating condition (although still have a life worth living), whereas if she waits a few months to conceive, her child will not have the condition. The woman chooses to conceive straightaway and gives birth to a child with the foreseen debilitating condition. Arguably, the child created has not been harmed by her mother's choice because, if the mother had waited and conceived later, this particular child would not have been born at all. Instead, a numerically different child would have been born because different originating gametes would have been involved in the conception. Hence the child cannot be said to have been harmed because she cannot be said to be worse off than she otherwise would have been. If the mother had waited, a (numerically) different child would have been born instead, for the children in each of the two scenarios are not numerically identical.

Two elements are required for Non‐Identity Problem to be properly understood. One is the gametic essentialist requirement; the other is an understanding of harm not in the sense of ‘violation of rights’, but in the sense of ‘worse off than otherwise would have been’.^19^ This is relevant to MRT in terms of whether or not one can consider living with mitochondrial disorders harmful (or their avoidance a benefit).

Claims to harm where different originating gametes are involved are thereby avoided under Non‐Identity Problem on the grounds that one cannot make the required comparison of relative levels of welfare between being created with or without the inherited genetic traits in question because any alternative selection of gametes would have resulted in a different person. Had different gametes been selected (for example, in such a way as to exclude those that might cause the mitochondrial genetic disorder), the resulting zygote would not have developed into that person and that person wouldn't have existed. Any claims to having been harmed in this way therefore come down to a choice between existence with a genetic disorder and not existing at all. As such, these choices cannot be deemed to harm the individual by making them ‘worse off than they otherwise would have been’ because the only alternative to that life is non‐existence.

The Non‐Identity Problem when applied to (some) reproductive technologies gives us the Non‐Identity Claim. This says:

When we use technique x this causes a (numerically) different person to be born, i.e. someone other than the person who would have been born if we had not used technique x.

We can now turn to ask whether the Non‐Identity Claim is true of MRT? If it is, children created using MRT cannot be said to have been harmed (or benefitted) by mitochondrial replacement, or at least it would not be harm in the usual comparative ‘harm‐to‐interests’ sense. We argue in what follows that the Non‐Identity Claim is *true of standard cases of MST but not true of standard cases of PNT.*

The question as to whether the Non‐Identity Claim holds for MRT depends on which method is chosen (PNT or MST), given that the methods differ in terms of when the mitochondrial replacement takes place. As the Origin View is central to the Non‐Identity Problem, then any selective choice of gametes in the MRT process prior to the fusion between sperm and egg would potentially be subject to this problem.^20^ With MST, manipulation of the maternal gamete is carried out prior to fertilization, whereupon it is fertilized with an available sperm. As the very process of manipulating the maternal gamete takes time, the sperm used to fertilize it (in standard cases) will be different from the sperm that would have fertilized it if the maternal gamete had not undergone manipulation. This means that a numerically different individual will be born than if MST had not been used.

The reason we have been referring to ‘standard’ cases is that MST is not *necessarily* identity‐affecting because, on our view, an egg which has its mitochondria replaced could remain numerically the same egg. However, there is good reason to think that *in practice* most cases of MST will be identity‐affecting because using MST will (nearly always) mean that a different spermatozoon will come to fertilize the egg. It is possible *in principle* for a particular spermatozoon to be identified at the outset as the one that the couple wishes to use (regardless of whether or not MST takes place), and in cases like that, identity could be preserved: for the very same egg and the very same spermatozoon would be used irrespective of whether MST takes place. In practice, however, it is extremely doubtful that the identification of a single spermatozoon in advance is something that will happen (not least because there is no clinical reason to do this). So, in any case where fertilization takes place by merely mixing numerous spermatozoa with the post‐MST egg, the chances of this egg being fertilized by the very same individual spermatozoon that would have fertilized it had MST not taken place must be miniscule. Identity‐preservation can only be guaranteed when the identification of a single spermatozoon in advance takes place. Accordingly, we hold that MST is nearly always identity‐affecting because, even if the egg is still the same egg after MST, there's a very high chance that it will be fertilized by a different spermatozoon.

With PNT, however, the intervention happens after fertilization, the gametes used are unaffected, and so the Non‐Identity Problem does not arise. This means that PNT is capable of benefiting or harming any child created in a straightforward ‘harm‐to‐interests’ sense. PNT could therefore be a beneficial treatment for a particular individual who would otherwise have suffered from debilitating mitochondrial disease. Given that one of the charges laid against MRT is that it is a reproductive technology whose only function is to allow a woman carrying certain mitochondrial genetic mutations to be genetically related to the child they gestate,^21^ this approach might answer those critics by establishing that the method of PNT is about improving the health of a particular child.^22^

## 5. Ethical Implications

We turn now to discuss the ethical implications for MST and PNT. As concluded in the previous section, MST and PNT are different in the following respect: the Non‐Identity Claim is true of (at least most) instances of MST; hence MST can best be classified as a kind of selective reproduction. It is not, however, true of PNT; so PNT is embryo‐modification or embryo‐therapy, rather than selective reproduction.^23^ This section asks what ethical implications this difference might have. Assuming that MST and PNT are equally safe, effective, and cost‐effective in all respects, does this difference matter ethically? We focus on two main differences. First, there is a suggestion that ‘at risk’ parents have stronger moral reasons to accept PNT than to accept MST because failing to use PNT (but not failing to use MST) could directly harm their future children. Second, we look briefly at the idea that objections to mitochondrial replacement based on concerns about eugenics and human dignity engage more strongly in the case of MST.

### 5.1 Identity, Harm and Parental Obligation

The Non‐Identity Claim says that, when we use MST, this causes a (numerically) different person to be born: that is, someone other than the person who would have been born if we hadn't used MST. Now consider the following example.

Two parents, who have an almost 100% chance of their child having a mitochondrial disorder if they conceive naturally, are offered MST to avoid their child having mitochondrial disease.^24^ If they decide not to use MST but conceive naturally, they will have Child A. If they decide to accept MST, they wouldn't have Child A, but a numerically different child, Child B. Imagine first that the parents go for unassisted conception, resulting in child A. Child A suffers from a life‐limiting mitochondrial disorder. When old enough, the circumstances of her conception are disclosed to her, including the fact that MST *could have been but was not* used. Although Child A may consider that her parents have harmed her by knowingly subjecting her to a mitochondrial disorder, it is clear that although they could have created a different embryo through MST and have had a child without the disorder, that child would have been Child B, not Child A. So, while Child A's life would have been better if she had been born without a mitochondrial disorder, that was not one of the options available. Rather, their choice was between *Child A with* and *Child B without* mitochondrial disorder. Child A cannot therefore reasonably claim that she has been harmed (made worse off) by her parents' decision, for the only available alternative was not a disorder‐free life but non‐existence. So provided that Child A does not feel, all things considered, that she would be ‘better off dead’, she cannot reasonably claim to have been harmed by her parents' reproductive decision.^25^

Generalizing from this, one important implication is that (unless the mitochondrial disease would be so bad that the child would be ‘better off dead’ – which may be true of *some* mitochondrial disorders) the rationale for using MST can't be to avoid harm to a particular child.^26^ The rationale for using MST must instead be one or more of the following. Firstly, it could be driven by ‘impersonal’ welfare considerations: specifically the thought that, when there's a choice between creating one of two (or more) possible future children, we should choose the one with the best expected quality of life. Thus faced with a choice between creating Child A (with a mitochondrial disorder) and Child B (without a mitochondrial disorder) the thought is that we should create Child B on the grounds that her expected quality (and length) of life will be greater. The underpinning principle at work here is what Parfit terms the ‘Same Number Quality Claim’, an idea echoed in a much later article by Julian Savulescu called ‘Procreative Beneficence’.^27^

A second possible rationale is the interests of the parents. Looking after a child with a mitochondrial disorder is challenging and, even if they would be good and loving parents to Child A, they may still prefer instead to raise a child without a serious genetic disorder. Finally, cost‐saving is another possible (although controversial) rationale for providing MST.

PNT is in a different position because the Non‐Identity Claim does not apply. Imagine a similar situation to the one just described but one in which the parents are offered PNT rather than MST. In this second scenario, their choice is between rejecting PNT, in which case Child A will be born *with* a mitochondrial disorder, and accepting PNT, in which case Child A will be born *without* a mitochondrial disorder. PNT is then in effect an attempt at a ‘pre‐emptive’ *cure* for mitochondrial disorder; one which happens at the embryonic stage.

This has major implications for the way in which the idea of *harm* can be deployed. Imagine that the parents decline the offer of PNT and that Child A is born with mitochondrial disease. As in the first scenario, when she is old enough to understand, the fact that PNT *could have been but was not* used is disclosed and she blames her parents for *harming* her for (what she sees as) knowingly subjecting her to this awful disease. The crucial difference in the PNT scenario is that in this case Child A is correct. Her parents may or may not have had good reasons for declining PNT, but – whatever their reasons – it cannot be denied that their choice harmed Child A. For, had they used PNT, she would be living without rather than with a mitochondrial disorder. Thus there is a strong *prima facie* harm‐avoidance rationale for offering PNT to prospective parents, and for those parents to accept it; one that is not present in the case of MST.

It may also be argued, because of this difference *vis‐à‐vis* harm, that parental ethical obligations are different, at least in strength, between the two cases. For PNT, their refusal to let Child A benefit from the procedure is in some respects like harming her during pregnancy by refusing to accept medication required to protect the fetus. In both cases (PNT and fetal medicine), Child A would (eventually) be harmed and – once she comes to be born – can blame her parents for decisions which harmed her, or at least which deprived her of benefit. So, if we think that prospective parents have a moral reason to refrain from harmful actions during pregnancy, and if we similarly think that they have a moral reason to consent to medical procedures needed by their fetus (provided that these are not unduly onerous or dangerous for the woman), then we may similarly feel they are under a strong obligation to accept PNT in order to protect Child A from harm.

Although we hold this view to be broadly correct, it is important to note some caveats. First, there are clearly differences between intervening at the embryonic stage and intervening during pregnancy. For example, medication accepted during pregnancy may affect the pregnant woman, whereas this is less likely in the case of PNT – so, in this respect, the case for accepting PNT may be stronger. Also, ‘gradualists’ may feel that, at least in late pregnancy, the fetus has a stronger moral claim on us to provide beneficial treatment than does an early embryo.^28^ So we do not claim that medication during pregnancy and PNT are identical: merely that they are similar in important respects. Moreover, parents and doctors may disagree about what's best for the (future) child and if parents declined PNT because they (reasonably) believed it on balance would do more harm than good then they would perhaps be less blameworthy than parents who decline the treatment for other reasons.

With MST, since we classify this as selective reproduction, the parents' ethical position here is closer to what it would be when considering practices like PGD and prenatal testing. In all such cases, a parental decision to intervene to avoid mitochondrial disorder would in effect be a decision to opt for Child B over Child A: choosing one possible future child over another. It may be that parents do have a moral obligation to make such a choice (‘Procreative Beneficence’ could underpin such a duty). That obligation, however, is less clear, more controversial, and/or perhaps weaker than the ‘child protection’ obligation that is in play in the PNT case.^29^

So, unless one's ethical position is strongly ‘impersonal’ and consequentialistic in nature (which is of course an option and not one we can conclusively refute here) it will be difficult to resist the conclusion that prospective parents' obligations to accept – and, for the same reasons, clinicians' and funders' obligations to provide – PNT are stronger than their obligations to provide MST (on the assumption, as we have said, that both are effective and safe).

### 5.2 Human Dignity and Eugenics

We briefly turn now to the idea that that concerns about eugenics and human dignity engage more strongly in the case of MST than in the case of PNT. If only one of the two MRT techniques is selective reproduction then the following argument only applies MST, not PNT:

… what is being proposed by the HFEA is not a form of therapy in which a person is being treated or cured for a disorder. Instead, it is making sure that certain persons are not brought into existence. This is a crucial difference since it then questions the equality in value and worth of every possible future person. Moreover, this equality of all existing and possible future human beings is one of the foundations of inherent human dignity.^30^

We do not make any claims here about the soundness or otherwise of arguments like MacKellar's, although we do note that such arguments have been extensively critiqued in the existing literature.^31^ Rather our suggestion is conditional and relative: *if* such arguments have any merit (and some people think that they do) then they apply *more forcefully* against MST than against PNT, because only the former is an instance of selective reproduction. As MacKellar says, ‘making sure that certain persons are not brought into existence’ seems especially problematic for those who take human dignity to be an important moral principle, as it could be taken to imply that not all (possible future) persons are ‘equal in value and worth’ and that some (possible future) persons are better, more worthy, candidates for existence than others.

Turning to eugenics, there are various different definitions of this contested concept but the common core which most of them share is that eugenics is the attempt to improve the human ‘gene pool’.^32^ On this definition, it looks as if mitochondrial replacement could be eugenic because it seeks, amongst other things, to improve the human ‘gene pool’ by reducing the prevalence of heritable mitochondrial disorders in the population. This is not necessarily an *objection* to mitochondrial replacement, because the same could be said of the existing widely accepted alternatives – such as refraining from having children or using an egg donor. Both of these are ways not only of having a child free from disorders, but also of improving the future ‘gene pool’ by avoiding the passing on of mitochondrial disorders to future generations. Furthermore, reducing the prevalence of mitochondrial disorders (be it technically eugenic or not) seems to be a pretty laudable aim and one that many would endorse. So it may be that in terms of their *aims* both MST and PNT are, in one sense, eugenic although it does not follow from this that either is unethical. Nor does it follow that these technologies are any *more* eugenics than existing methods of selective reproduction (abstinence, adoption, egg donation, *etc.*).

But are MST and PNT *equally* vulnerable to accusations of eugenics? Perhaps there are reasons for thinking that such charges are weaker in the case of PNT. One is parental motives. In the case of PNT, as we have seen, the parents can be – and probably should be – focussed on protecting a determinate child from harm, on ensuring that he or she gets access to ‘pre‐emptive treatment’, which is what PNT is. So there's a good chance that the parents' reasons for seeking PNT will have nothing to do with successive generations; rather their concern is mainly with preventing *one particular child* from suffering. In the case of MST, though, the parents won't be able to appeal to this child‐protection rationale. Their reasons for acting must be more ‘impersonal’, more ‘selective’; they want to avoid having *this* child (with mitochondrial disorder) and want instead to have *that* child (without mitochondrial disorder). As before, the parents in this situation won't necessarily have much interest in *population* health, but it could nonetheless be argued that their desire to reproduce selectively is more *like* eugenics than is the PNT parents' desire to *cure* their child. Alternatively, one might argue that such MST‐selective decisions are constitutive of and a means of achieving a wider eugenics; whereas the same can't be said of the PNT parents for whom any eugenic effects are more like the side‐effects of medical treatment.

## 6. Conclusion

At first glance, the two versions of mitochondrial replacement being developed look to be the same in all but some particular technical respects: one (MST) replaces the mitochondria in eggs, the other (PNT) replaces the mitochondria in embryos. However, we have argued that, contrary to appearances, MST and PNT are different in fundamental respects. In particular, PNT is a treatment which is attempting ‘pre‐emptively’ to *cure* a person without affecting his or her identity. Thus, PNT is like, or is even a form of, gene therapy. MST, on the other hand, is a form of selective reproduction and has more in common with pre‐implantation genetic diagnosis and pre‐natal screening than it does with gene therapy.

What ethical implications does this have? First, all other things being equal, parents offered PNT have a stronger obligation to accept it than do parents offered MST. This is because parents who decline PNT could be said to be harming their future child, whereas parents who decline MST will merely be failing to ‘substitute’ one possible future child with another (failing to ‘de‐select’ a possible future child with a mitochondrial disorder). Second, because MST is a form of selective reproduction rather than therapy, concerns about eugenics and human dignity may engage more strongly in the case of MST than in the case of PNT.

Therefore, if (and this is a big ‘if’) MST and PNT were to turn out to be equally safe and effective, we would have some ethical reason to prefer PNT.

## Footnotes

1
For example, a number of ethical concerns have been raised about the use of MRTs, including: the health risks of having children with three progenitors; the use of sex selection in conjunction with MRTs; the permissibility of MRT research on embryos; severing the mitochondrial genetic tie between intending mother and child; and whether MRTs constitute ‘germ line modification’ and/or ‘genetic modification’; A. Bredenoord & P. Braude . Ethics of Mitochondrial Gene Replacement: From Bench to Bedside. Br Med J 2011; 342: 879; Nuffield Council on Bioethics . Novel Techniques for the Prevention of Mitochondrial DNA Disorders: An Ethical Review. London: Nuffield Council on Bioethics; 2012; F. Baylis . The Ethics of Creating Children With Three Genetic Parents. Reprod BioMed Online 2013; 26: 531–534.2360824510.1016/j.rbmo.2013.03.006

2

A.L. Bredenoord . et al. Dealing With Uncertainties: Ethics of Prenatal Diagnosis and Preimplantation Genetic Diagnosis to Prevent Mitochondrial Disorders, Hum Reprod Update 2008; 14: 83–94.1805613310.1093/humupd/dmm037

3

Nuffield Council on Bioethics . *op. cit* 1, p. 25; A.M. Schaefer , et al. Prevalence of Mitochondrial DNA Disease in Adults. Ann Neurol 2008; 63: 35–39.1788629610.1002/ana.21217

4
For example, it has been reported that countries such as the UK, Sweden, and Australia have experienced a scarcity of donor eggs; A.S. Svanberg . Characterization of Potential Oocyte Donors in Sweden. Hum Reprod 2003; 18; 2205–2215; 1450784510.1093/humrep/deg398 I. Torjesen . Fertility Regulator Tells Clinics to Treat Egg And Sperm Donors Better to Boost Numbers. Br Med J 2005; 344; 2249195810.1136/bmj.e2594 J. Medew . 
*The Age – IVF Deal Sees American Eggs Heading Down Under*
. 2013 Available at: http://monashivf.com/the‐age‐ivf‐deal‐sees‐american‐eggs‐heading‐down‐under/ [cited 2014 Sept 20].

5

UK Department of Health . 
*Mitochondrial Donation: A Consultation on Draft Regulation to Permit the Use of New Treatment Techniques to Prevent the Transmission of a Serious Mitochondrial Disease From Mother to Child*
. 2014 Available at: https://www.gov.uk/government/uploads/system/uploads/attachment_data/file/285251/mitochondrial_donation_consultation_document_24_02_14_Accessible_V0.4.pdf [cited 2014 Sept 20].

6
Researchers are currently unsure whether a small amount of the unhealthy mitochondria from the discarded embryo is likely to be inadvertently transferred to the new embryo during the process of transferring the pronuclei. Nuffield , *op. cit.* 1, p. 67.

7

Human Fertilisation and Embryology (Mitochondrial Donation) Regulations 2015 (HFER 2015). Available at: http://www.legislation.gov.uk/ukdsi/2015/9780111125816/pdfs/ukdsi_9780111125816_en.pdf [cited 2015 May 11].

8
Some, such as Reinhardt et al., have raised concerns about the possibility of health complications arising from ‘mismatching’ between nuclear DNA and the donor's mtDNA. The HFEA has responded to these concerns by stating that the available evidence on any possible ‘mismatches’ resulting from MRT use ‘did not show cause for concern’ and that the HFEA would continue to review evidence on MRTs. Previous statements from Nuffield Council on Bioethics support the ongoing review of evidence relating to this area of mitochondrial biology: ‘further studies using human embryos and research with animals will be important to disclose any potential for the manipulation of embryos and gametes to cause chromosomal or epigenetic problems’, Nuffield , *op. cit.* 1, p.66; K. Reinhart , D. Dowling & E. Morrow . Mitochondrial replacement, evolution and the clinic. Science 2013; 341: 1345–1346; 2405229410.1126/science.1237146 Human Fertilisation and Embryology Authority (HFEA). 2013; HFEA statement regarding the Klaus Reinhardt et al. Science paper ‘Mitochondrial replacement, evolution, and the clinic’. Available at:http://www.hfea.gov.uk/8178.html?size=large [Accessed 2015 April 27]; Human Fertilisation and Embryology Authority (HFEA). 2015 Statement on mitochondrial donation. Available at: http://www.hfea.gov.uk/9606.html [Accessed 2015 April 27]. It is beyond the scope of this article to discuss this contended area of science in further detail.

9
We will leave to one side for now further philosophical problems pertaining to the coherence of this conception of identity, such as have been raised by P. Geach . Reference and Generality. 3rd ed. Ithaca, NY: Cornell University Press; 1980; D. Lewis . On the Plurality of Worlds. Oxford: Blackwell; 1986.

10
More precisely, as we shall discuss, it is the originating gametes that have the central role in a causal account of our identity and thereby serve rigidly to designate the same individual across all possible worlds.

11

S. Kripke . Naming and Necessity. Oxford: Blackwell; 1980; D. Parfit . Reasons and Persons. Oxford: Oxford University Press; 1984.

12
Note that this is a necessary property but not a sufficient one for the identity of a human being. This is due to cases such as monozygotic twins, where both have the same originating gametes. However, the important point for the identity arguments is that difference in originating gametes must therefore indicate difference in identity.

13

J. Feinberg . Wrongful Life and the Counterfactual Element in Harming. Soc Philos Policy 1987; 4(1): 145–178; 1165878310.1017/s0265052500000467 M. Hanser . Harming Future People. Philos Public Aff 1990; 19: 47–70; 11651965 D. Velleman . Persons in Prospect. Philos Public Aff 2008; 36: 221–288; A. Wrigley . Genetic Selection and Modal Harms. Monist 2006; 89(4): 505–525; A. Wrigley . Harm To Future Persons: Non‐Identity Problems and Counterpart Solutions. Ethical Theory Moral Pract 2012; 15: 175–190.

14
There remain additional concerns beyond the scope of this paper, such as whether adding a ‘third parent’ gene into someone's germ line is a harm or benefit in some non‐person affecting way, such as by being a societal good or ill.

15

Parfit . *op. cit.* 11, p. 352.

16
For a critical discussion of the role of the Origin View, see P. Mackie . How Things Might Have Been: Individuals, Kinds, and Essential Properties. Oxford: Oxford University Press; 2006.

17
The necessity of origins thesis is not universally accepted, although it is widely endorsed, and even famous detractors of it such as Lewis , *op. cit.* 9, and Mackie *, op. cit.* 16, recognize the importance of a match of origins in determining the identity of an individual.

18

Parfit , *op. cit.* 11, pp. 358–359.

19
A discussion of concepts of ‘harm’ can be found in J. Feinberg . Harm to Others: The Moral Limits of the Criminal Law *.* New York: Oxford University Press; 1984. A summary can also be found in S. Wilkinson . Bodies for Sale: Ethics and Exploitation in the Human Body *Trade*. London: Routledge; 2003 59–60.

20
It should be emphasized that the manipulation or damage of gametes is not subject to non‐identity arguments in the same way unless that process itself changes the identity of the gamete(s).

21
The basis for this claim is that there are other reproductive options, such as surrogacy or IVF using donated eggs, that are open to parents but which wouldn't allow the woman to be genetically related to the child; Bredenoord & Braude , *op. cit.* 1, pp. 87–89.

22
Although concerns may be raised about the possibility of epigenetic interactions having an impact on identity, they would not be specifically applicable to cases concerning numerical identity. Epigenetic interactions would only occur after MRT has taken place, so won't affect the originating gametes and, consequently, they would be unable to affect an individual's numerical identity. Epigenetics may, however, be of genuine concern where other senses of identity are relevant, such as an individual's qualitative identity. In these cases, the development of qualitative properties through epigenetic interactions could potentially be identity‐affecting. This is an interesting additional consideration but, as highlighted in section 3, discussion of these other senses of identity is beyond the scope of this paper.

23
To‐date, there has been some debate about whether or not MST and PNT should be considered ‘genetic modification’. Baylis has argued that MRTs constitute genetic modification because they involve exchanging the mitochondrial genes that the intending mother has supplied with the mitochondrial genes of a donor. Others, such as the UK Department of Health, also recognize that the use of MRT involves the replacement of one mitochondrial genome with another. However, it argues that the mitochondrial genome is only being entirely replaced, rather than having any one part, or parts, of its genetic constitution modified. Therefore, in contrast to Baylis, the UK government has instead opted to refer to MRTs as a form of ‘germ‐line modification’ rather than ‘genetic modification’; UK Dept. of Health , *op. cit.* 5, p. 12; Baylis , *op. cit.* 1, p. 533; Nuffield Council on Bioethics , *op. cit.* 1, pp. 57–58; S. Barber & P. Border . Mitochondrial Donation. 
*House of Commons Library*
; 2015 SN/SC/6833: 19–21.

24
We are here referring to homoplasmic mutations. While, it is generally understood to be true that anyone inheriting a homoplasmic mtDNA mutation will suffer from it, there is a very small chance that a person could potentially be a carrier and not suffer from the harmful effects of the mutation; Bredenoord et al., *op. cit.* 2, pp. 83–94.

25

S. Wilkinson . Choosing Tomorrow's Children. Oxford: Oxford University Press: 2010 4–7.

26
Some mitochondrial disorders manifest in particularly terrible ways and it might be argued that living with such disorders is worse than not living at all. For example, Leigh's Syndrome can cause death to a child within the first few years (sometimes months) of life. Symptoms during that time may include the failure of muscular systems (including cardio‐respiratory systems and the ability to swallow), failure of visual systems, and damage to the nervous system; Nuffield Council on Bioethics , *op. cit.* 1, pp. 27, 73–74.

27

Parfit , *op. cit.* 11; J. Savulescu . Procreative Beneficence: Why We Should Select the Best Children. Bioethics 2001; 15: 413–426.1205876710.1111/1467-8519.00251

28

R. Dworkin . Life's Dominion: An Argument About Abortion and Euthanasia. London: Harper Collins 1993; R. Scott . Rights, Duties and the Body: Law and Ethics of the Maternal‐fetal Conflict. Oxford: Hart Publishing: 2002 43–56.

29

Savulescu , *op. cit.* 27, S. Sheldon & S. Wilkinson . Termination of Pregnancy for reason of foetal disability: Are there grounds for a special exception in Law? Medical Law Review 2001; 9: 85–109; 1277892810.1093/medlaw/9.2.85 S. Wilkinson . Prenatal Screening, Reproductive Choice, and Public Health 2015; 29: 26–35.10.1111/bioe.12121PMC431589725521971

30

C. MacKellar . Should Persons Affected by Mitochondrial Disorders Not Be Brought Into Existence? 
*Bionews*
 2014; 736 Available at: http://www.bionews.org.uk/page_385343.asp [cited 2014 Sept 20].

31
See, for example, Wilkinson *op. cit.* 25, pp. 148–185.

32

R. Chadwick . *Genetics and Ethics* *.* In: CraigE., editor. The Routledge Encyclopaedia of Philosophy. London: Routledge; 1998.

